# Non-Traumatic Pneumocephalus and Sub-Dural Empyema as a Complication of Chronic Sinusitis

**DOI:** 10.7759/cureus.5202

**Published:** 2019-07-22

**Authors:** Sidra Saleem, Arsalan Anwar, Hobab Aslam, Pulwasha M Iftikhar, Owais Ur Rehman

**Affiliations:** 1 Neurology, University of Toledo, Toledo, USA; 2 Neurology, University Hospitals Cleveland Medical Center, Cleveland, USA; 3 Pediatrics, Sindh Medical College, Karachi, PAK; 4 Obstetrics and Gynecology, St. John's University, New York, USA; 5 Internal Medicine, Civil Hospital Karachi, Dow University of Health Sciences, Karachi, PAK

**Keywords:** pneumocephalus, chronic sinusitis, subdural empyema, intracranial complications

## Abstract

Infectious sinusitis is extremely common in children, and persistent infection can lead to many complications. The most dangerous and commonly reported complications are intracranial. These intracranial complications include pneumocephalus, cerebral abscess, subdural empyema, meningitis, cellulitis, orbital abscess, and cavernous sinus thrombosis. Pneumocephalus is the presence of air in the cranium and sometimes it can lead to intracranial infection and localized pus collection in the potential space between meninges. Herein, we report a case of a 12-year-old girl who presented to a pediatric emergency in a confused and disoriented state. The cerebrospinal fluid (CSF) analysis provided a picture of bacterial meningitis, but her CT scan showed pneumocephalus and subdural empyema. This case report will help clinicians overcome this diagnostic challenge using the appropriate imaging and treatment modalities to prevent neurological sequelae.

## Introduction

Infectious sinusitis is extremely common with the incidence of 15-40 episodes per 1000 patients per year [1]. It is usually viral but can be complicated by a bacterial infection with an incidence of 5% to 8%. Persistent sinusitis for more than 12 weeks causes chronic sinusitis, which if left untreated can lead to serious complications, with the most dangerous and commonly reported are neurological complications from intracranial and orbital spread of disease. Incidence of morbidity and mortality due to intracranial complications has decreased from 80% in 1999 to 30% in 2019 because of early diagnosis and better antibiotic coverage [2].

The most frequently reported intracranial complications are pneumocephalus, cerebral abscess, subdural empyema, meningitis, cellulitis, orbital abscess, and cavernous sinus thrombosis. Pneumocephalus (PNC) is defined as the presence of air in the cranium due to the connection between the intra-cranial and extra-cranial space. It can be either traumatic or non-traumatic. Traumatic pneumocephalus is more common and occurs due to head and facial injury, as a complication of surgery and invasive procedures of otorhinolaryngology. Non-traumatic pneumocephalus is uncommon, but it can be due to neoplasm of the skull, Valsalva maneuver, adjacent air sinus infection, and post-radiation [3]. Subdural empyema is a collection of pus between the dura mater and the arachnoid membrane [2]. It is most commonly caused by a frontal sinus infection or otogenic infection. Herein, we report a unique case with clinical signs mimicking meningitis, but no growth of organisms was seen on cerebrospinal fluid culture. Incidental pansinusitis and pneumocephalus were found on CT scan that led to subdural empyema.

## Case presentation

A 12-year-old girl presented to a pediatric emergency with confusion and disorientation. According to her mother, she suddenly developed high-grade fever followed by headache and projectile vomiting. She had no significant past medical history except on and off a cold since the age of five years. On examination, her blood pressure was 108/57 mmHg, her pulse was 130/minute, and her respiratory rate was 40/min. Her O_2_ saturation was 82%. She was non-responsive, having a Glasgow Coma Scale (GCS) of 8/15. There was no neck stiffness or signs of meningeal irritation. Motor examination showed no deficit with normal tone and power of the upper and lower limbs. Her knee and ankle reflexes were 2+ and the Babinski reflex was negative. On the basis of history and clinical examination, meningitis was suspected and cerebrospinal fluid sample (CSF) was sent for the detailed examination and culture and sensitivity (C/S). CSF analysis showed bacterial meningitis, WBC count of 297 (neutrophils: 95% and lymphocytes: 5%), protein 124, and glucose 52. Empirical treatment with antibiotics was started. Intravenous ceftriaxone 1.5 g twice daily and vancomycin 800 mg IV 8 hourly was given. She was treated as a case of bacterial meningitis, but on the fourth day of hospitalization, CSF culture and sensitivity report came negative.

**Figure 1:**
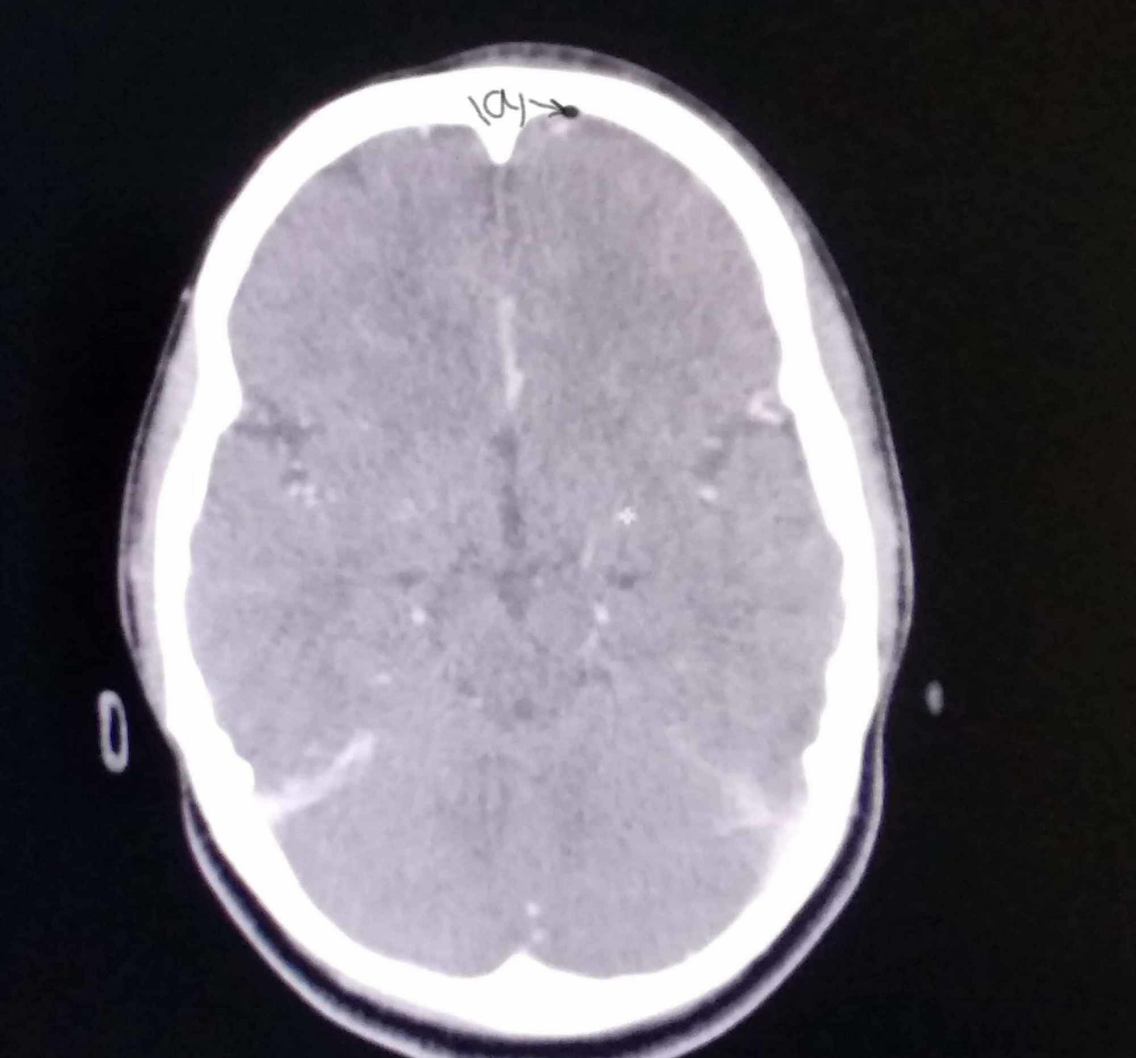
Axial CT scan, marked (a) is pneumocephalus in the frontal region

**Figure 2:**
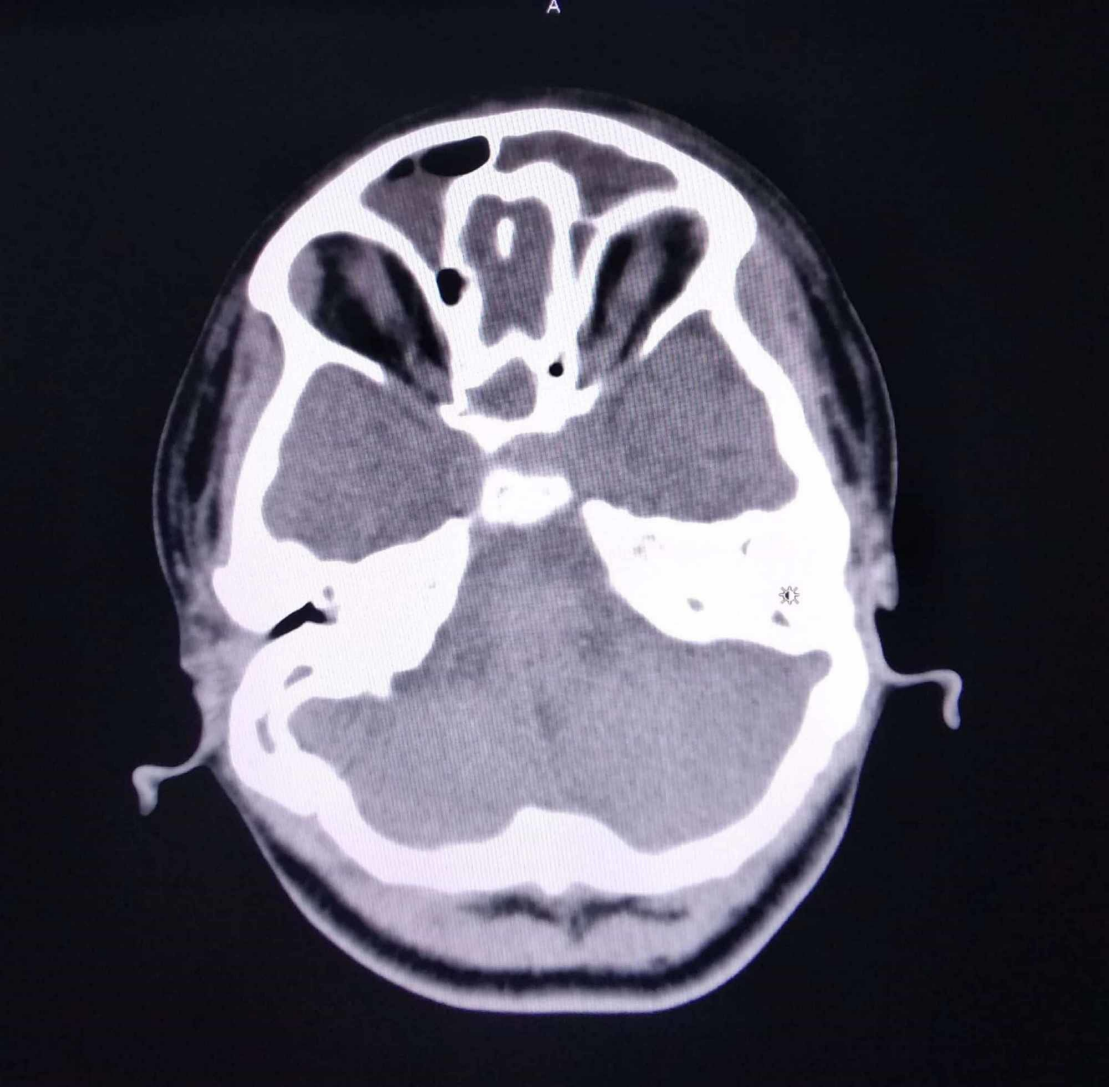
Axial CT scan, showing pansinusitis

CT scan was performed and pneumocephalus was observed in the right frontal region (Figure 1) and there was an incidental finding of pansinusitis with sclerotic, thickened bone and opacification of sinuses (Figure 2). Her CT scan of the head showed signs of chronic sinusitis and pansinusitis. This finding brought us to the conclusion that this chronic sinusitis may be responsible for pneumocephalus on CT scan by eroding the bone and false-positive CSF D/R of bacterial meningitis.

**Figure 3:**
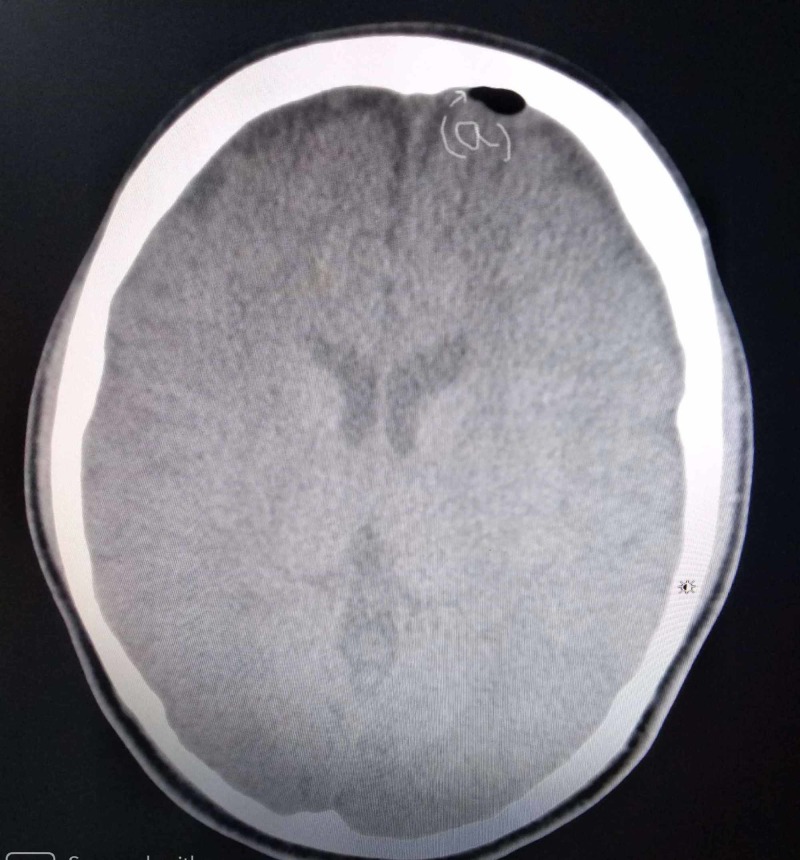
Axial CT scan, showing the increased size of pneumocephalus

**Figure 4:**
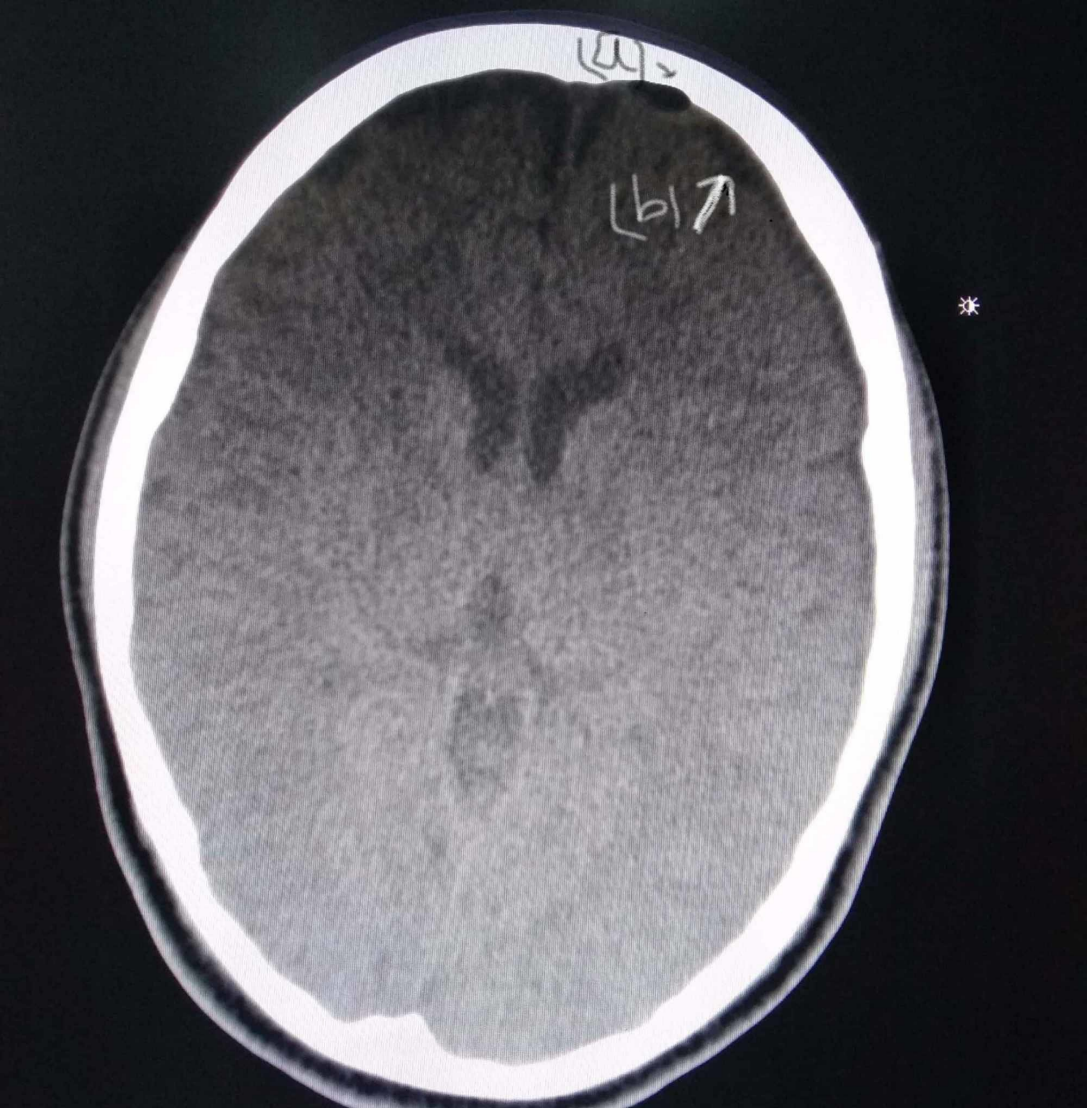
Axial CT scan; (a) pneumocephalus and (b) subdural empyema

During her hospital stay, she showed some symptomatic improvements to antibiotics. But after six days, her GCS started to deteriorate again (7/15) with high-grade fever spikes and an apparent swelling of the forehead. Her CT scan was repeated, which showed an increase in the size of pneumocephalus (Figure 3) with subdural empyema (Figure 4).

Immediate neurosurgical intervention was required, and surgical drainage was done for subdural empyema. On the second day of surgical drainage, the patient improved clinically with GCS 15/15 and her fever also subsided. The patient was stable and discharged with a prescription of oral antibiotics, nasal saline spray, and nasal decongestants. She was seen in follow-up after two and four weeks and her symptoms improved significantly.

## Discussion

Sinusitis has two types, acute and chronic. Acute sinusitis lasts less than 12 weeks and it is usually caused by rhinoviruses, parainfluenza and influenza virus. Chronic sinusitis lasts more than 12 weeks and it is usually caused by anaerobes, gram-negative bacteria, *Staphylococcus aureus*, and fungi [4].

Appropriate treatment of sinusitis is necessary because it can cause various dangerous complications either due to the direct extension of the infection to surrounding structures or tissue plane or due to persistent infection [5-6]. Due to chronic sinusitis, intracranial complications may present as a serious manifestation. These are localized cerebritis such as cerebral abscess, subdural empyema, epidural empyema, and cavernous sinus thrombosis [7-8]. Infected thrombi may form within the vessels and can erode the bone causing osteomyelitis. This can lead to fistula formation and bone tissue loss, which later on fills with granulated tissue. Septic marrow thrombophlebitis is one of the dreadful complications in the posterior wall of the frontal sinus [9-10]. The intracranial complications and symptoms are summarized in Table 1.

**Table 1:** Complications of chronic sinusitis

Complication	Symptoms
Suppurative intracranial complications - Cerebral abscess - Subdural empyema - Epidural empyema	Fever, headache, nausea, vomiting, altered mental status, focal neurologic deficits, seizures, meningismus
Meningitis	Fever, headache, neck stiffness, nausea, vomiting, altered mental status
Preseptal cellulitis	Eyelid edema and erythema
Orbital cellulitis	Chemosis, proptosis, orbital pain, ±restricted extraocular movements
Orbital abscess	Severe proptosis, chemosis, restricted extraocular movements, visual loss
Cavernous sinus thrombosis	Orbital pain, headache, proptosis, visual loss, palsies of cranial nerves III, IV, VI, V1, and V2, picket fence fevers; often bilateral

Even with an intact osseous wall, white matter alterations are present as dura adheres tightly to the bone. Therefore, we cannot exclude brain abscess even with the intact sinus wall. Although cerebral cortex and white matter have been resistant to inflammation for a longer period, alterations occur in white matter due to continuous infection and involvement of cancellous diploe of osseous walls of the cranium [11]. Few cases have been reported in the literature describing intracranial complications due to chronic sinusitis. Benevide et al. reported a case of a 14 year old who initially presented with the dental infection which deteriorated later and CT subdural empyema and bony erosion, the same pathology of untreated chronic sinusitis leading to dangerous sequelae as in our case [12]. Pishbin et al. reported a case of a 51-year-old female with disseminated pneumocephalus with the bony defect of sphenoidal sinus as the same pathology of PCN of our case but there was an involvement of frontal sinus [13]. The lack of cases in the literature reporting PNC due to chronic sinusitis emphasized the significance of CT scan and face X-ray in patients with fever, headache, and on and off cold. Therefore, more case reports and case series are needed to set the standard approach for these patients.

## Conclusions

This case report suggests that pediatric patients have a higher chance of intracranial complications from chronic bacterial sinusitis. PNC and subdural empyema are still encountered with alarming morbidity despite antibiotic use due to delayed diagnosis. Its variable presentation can lead to delayed diagnosis in the pediatric group as mimicking other brain infections. The only way to overcome this diagnostic challenge is to have significant knowledge of chronic sinusitis and its complications and using the appropriate imaging and treatment modalities to prevent neurological sequelae.
